# Double Guard Efficiency and Safety—Overcoming Resistance to Immunotherapy by Blocking or Stimulating Several Immune Checkpoints in Non-Small Cell Lung Cancer Patients

**DOI:** 10.3390/cancers15133499

**Published:** 2023-07-05

**Authors:** Ewa Kalinka, Kamila Wojas-Krawczyk, Paweł Krawczyk

**Affiliations:** 1 Department of Oncology, Polish Mother’s Memorial Hospital—Research Institute, 93-338 Łódź, Poland; 2 Department of Pneumonology, Oncology and Allergology, Medical University of Lublin, 20-090 Lublin, Poland; kamilawojas@wp.pl (K.W.-K.); krapa@poczta.onet.pl (P.K.)

**Keywords:** non-small cell lung cancer, immunotherapy, resistance, immune checkpoints

## Abstract

**Simple Summary:**

As immunotherapy is one of the leading treatment approaches in non-small cell lung cancer, immunotherapy primary or secondary resistance mechanism need to be deeply understood. Moreover, it is important to know how to avoid or overcome resistance – both have been widely discussed in the manuscript based on known trials’ results.

**Abstract:**

Immunotherapy is one of the leading systemic therapies in non-small cell cancer (NSCLC) patients, but it is not effective in an important proportion of them due to primary or secondary resistance mechanisms. Clinicians do not have the tools to predict immunotherapy resistance, and thus, many patients still fail initial treatment. One of the scientific concepts to avoid resistance and/or offer the patient effective salvage second-line treatment is the dual immunologic checkpoint blockade. We aimed to review published and available data on combination immunotherapy in terms of mechanisms, efficacy, and safety data on many different dual blockades. We discussed the potential of combined CTLA-4 (Cytotoxic T Lymphocyte Antigen 4), PD-1 (Programmed Death 1) or PD-L1, TIGIT, LAG-3, TIM-3, macrophage leukocyte immunoglobulin-like receptor B2 (LILRB2/ILT4), S15-mediated immune suppression (SIGLEC-15), CD137, ICOS, and OX40 inhibitors in NSCLC treatment.

## 1. Introduction

Immunotherapy has been widely used in patients with non-small cell lung cancer (NSCLC). It is used in adjuvant treatment, in consolidation after chemoradiation, and in the therapy of patients with advanced/metastatic cancer. In the latter group of patients, immunotherapy is used in the first line of treatment as a monotherapy or in combination with chemotherapy, while it is used only as a monotherapy in the second line of treatment.

The expression of PD-L1 (programmed death ligand 1) on tumor cells is the predictive factor to immunotherapy or chemoimmunotherapy response. In some types of cancer, tumor mutation burden (TMB), microsatellite instability (MSI), and mismatch repair deficiency (dMMR) are also immunotherapy efficacy predictors. However, these factors are imperfect. Many NSCLC patients experience early, primary resistance to immunotherapy due to the initial presence of cancer cells that are resistant to treatment. Secondary resistance, which occurs after several months of treatment, has also been reported. This involves the selection of a unique clone of tumor cells that are resistant to immunotherapy that occurs among clones sensitive to treatment. The generation of new clones of treatment-resistant cancer cells (not present at therapy initiation) is associated with acquired resistance to immunotherapy [1].

Yang et al. identified 22 prospective studies which evaluated the efficacy and safety of retreatment with immune checkpoint inhibitors in 1865 patients with solid tumors. Retreatment was used due to disease progression after the use of CTLA-4 (Cytotoxic T Lymphocyte Antigen 4), PD-1 (Programmed Death 1), or PD-L1 inhibitors. A response to anti-CTLA-4 retreatment occurred in 12–23% of patients, and a response to anti-PD-1 retreatment occurred in 22–36% of patients (seven trials in total). A total of 13 studies evaluated anti-PD-L1 retreatment, and the overall response rate (ORR) was 5–53%. However, most of the studies concerned melanoma patients, and only 66 NSCLC patients were included in the clinical trials. In the KEYNOTE-10 and KEYNOTE-024 studies, 26 advanced NSCLC patients received retreatment with pembrolizumab due to progression after completing second or first-line pembrolizumab therapy. The ORR was 43% in the first study and 33% in the second trial. In the ATLANTIC study, durvalumab retreatment was applied in 40 patients with progression after completing 1-year durvalumab third-line treatment. The effectiveness of this procedure has not been reported. These studies included patients who had progressed a few months after the end of immunotherapy. They proved that tumor cells may remain sensitive to immunotherapy if it was previously effective. However, patients with progression during immunotherapy were not included in these studies, and this group of patients will be the subject of this review [2,3].

At present, it is not possible to predict the moment of immunotherapy resistance based on immunological or genetic tests. However, methods to overcome treatment resistance are emerging. One of them is combining two or more immune checkpoint inhibitors (ICIs) that act on different immune checkpoints. Another method is the addition of an agonist of costimulatory molecules to the ICIs. The mechanism of action of the simultaneous blockade of several immune checkpoints is explained in detail in the next section of the article.

Moreover, a point of major concern when double immunotherapy is initiated is the patients’ safety, with a lot of interest in grade 3–4 treatment-related adverse events (TRAEs) as well as those leading to death or permanent treatment discontinuation. Thus, we have reviewed the available data on published, ongoing, and initiated trials, which should help clinicians to make practical choices considering the mechanism of action, efficacy, as well as safety data on combinations.

## 2. Simultaneous Blockade of Several Immune Checkpoints—Mechanism of Action

An effective immune response is a particularly complex interaction between the immune system’s cellular elements and the cytokines released by them. The basic communication between the cells is mostly receptor contact, which is extremely important in provoking anti-tumour activity [4,5,6]. The interaction between antigen-presenting cells (APC) and T cells, defined as an immune synapse, takes place in a peripheral lymph node. Immune synapse formation is a necessary step to induce a specific immune response. Subsequently, the recognition of the tumor antigen by T lymphocytes in the MHC (Major Histocompatibility Complex) context occurs in the tumor microenvironment. This step is crucial for final T cell activation and consecutive tumor cell destruction [4,5,6].

The precise receptors’ interactions are the primary mechanism of intercellular communication. Therefore, for many years, modern immunotherapy has been focused on modulating the immune response by blocking or activating exact molecules to enhance and boost specific antitumor responses [4,5,6,7]. Two types of surface molecules can be distinguished on the immune cells, particularly on T cells. First are the molecules responsible for transmitting the signal that activates the lymphocyte, which include CD28, CD27, OX40, GITR (Glucocorticoid-Induced TNF Receptor), and CD137 (molecules defined as “positive” immune checkpoints). Stimulation of those molecules increases T lymphocytes’ proliferation, differentiation, and activation, and finally positively stimulates the development of an immune response. The second group of molecules includes those responsible for transmitting the lymphocyte-inhibiting signal, which include the following molecules: CTLA-4, PD-1, ICOS (Inducible T Cell Costimulator), LAG-3 (Lymphocyte-Activation Gene 3), TIM-3 (T Cell Immunoglobulin and Mucin-domain containing-3), and TIGIT (T Cell Immunoreceptor with Immunoglobulin and ITIM Domain) (molecules defined as “negative” immune checkpoints). Their stimulation leads to the functional exhaustion of T lymphocytes, progressive loss of their functions, inhibition of the immune response, and finally, immunosuppression [4,5,6,7,8].

In summary, a new approach of immunotherapy aims not only to block a single molecule with specific antibodies (anti-PD-1, anti-PD-L1, anti-CTLA-4), but rather to act on two different checkpoint inhibitors. It is well understood that the immune response’s exhaustion can be provoked by the increasing activity of negative checkpoints other than PD-1 or CTLA-4. Therefore, the combination approach can be particularly eagerly used at the time of patients’ progression in immunotherapy. Moreover, the effectiveness of immunotherapy stimulating “positive” immune checkpoints showed synergic activity when administrated with “negative” checkpoint inhibitors as well as when it was tested as monotherapy [4,5,6,7]. The exact mechanism of action of chosen molecules and their function in regulating immune response will now be discussed.

## 3. Synergistic Role of Anti-PD-1 and Anti-CTLA-4 Antibodies

The idea of using two different ICIs in cancer patients is based on the phenomenon that anti-PD-1 and anti-CTLA-4 antibodies can stimulate immune response at different spatial and temporal levels [4,9]. Antibodies that block CTLA-4 molecule work in the induction phase of the immune response, unblocking the activity of T lymphocytes at an early stage of activation in peripheral lymph nodes, where lymphocytes learn to recognize antigens. This is a critical point to stimulate further activity of T lymphocytes. The CTLA-4, instead of the CD28 molecule (the main costimulatory molecule), binds with CD80 and CD86 molecules on APC, which inhibits the proliferation and activation of T helper and cytotoxic lymphocytes. Furthermore, this interaction leads to the exfoliation of CD80 and CD86 molecules from the surface of antigen-presenting cells, causing their non-functionality. High expression of CTLA-4 on T lymphocytes also induces the intracellular FoxP3 (Forkhead box P3) protein, resulting in the transformation of these cells into T regulatory lymphocytes [4,9,10]. These lymphocytes play an important immunosuppressive role in relation to other immune cells. Antibodies that block PD-1 molecules are mostly active in the tumor microenvironment, in the executive (effector) phase of the immune response restoring T cells’ functioning at a late stage of their activity. For these reasons, the combination of two different immune checkpoint inhibitors results in a very effective activation of specific cytotoxic T cells. In this way, we obtain a more comprehensive approach to stimulating the immune system. However, as it was shown, CTLA-4-positive cells are responsible for ensuring immune tolerance and balance between action and suppression of immune cells. Therefore, unlocking their peripheral activity (by anti-CTLA-4 antibodies) can result in immunological side effects of immunotherapy, with autoimmune inflammation characteristics. However, it is definitely desirable to block the regulatory T lymphocyte function in the tumor microenvironment. Furthermore, stimulating their ADCC (Antibody-Dependent Cell-Mediated Cytotoxicity) activity may also contribute to increasing the effectiveness of immunotherapy directly in cancer tissue. The scientists had proposed a very interesting chemical correction of Fc fragment to reduce the peripheral activity of unblocked T lymphocytes and, at the same time, maintain their anti-cancer activity directly in tumor tissue. Nonfucosylated modification in the Fc region of anti-CTLA-4 antibody increases intratumoral T regulatory depletion, enhances T effector function, as well as improves ADCC by increasing anti-CTLA-4 antibody binding affinity to CD16 molecules (Fc receptor) on NK cells [4,9,11].

## 4. Negative Immune Checkpoints—LAG-3, TIM-3, TIGIT and News

Lymphocyte-activation gene 3 is a protein that belongs to the co-inhibitory receptors [12,13,14]. Interaction of LAG-3 with its ligand—major class II histocompatibility complex (MHC II)—on APC leads to lymphocyte exhaustion and suppression of the immune response [15]. LAG-3 can also be stimulated by soluble actors (e.g., galectin-3) secreted by apoptotic tumor cells. It is postulated that LAG-3 inhibits proliferation and reduces cytotoxic proteins (granzyme and granulysin) as well as cytokine (IL-2) production by T effector cells. LAG-3 expression has also been described in other immune cells, including B cells, NK cells, and unconventional T cells [16]. LAG-3 expression contributes to the immune escape mechanism of tumors and is often observed in tumor-infiltrating lymphocytes during cancer progression. It is postulated that increased LAG-3 expression is a very early marker of T lymphocyte exhaustion. Recurrently, simultaneous co-expression of other co-inhibitory molecules is observed [12,13,14].

T cell immunoglobulin and mucin-domain containing-3 is a protein targeted by carcinoembryonic antigen cell adhesion molecule 1 (CEACAM1) and phosphatidylserine (PtdSer) [17,18,19]. Additionally, two soluble ligands: galectin-9 and high-mobility group protein B1 (HMGB1), produced by dying cancer cells, can also act with TIM-3. All these interactions trigger the immunosuppressive pathways in inhibiting adaptive immune response, e.g., stimulating Th1 cell apoptosis and inhibiting T cell response in tumors. Additionally, the interaction of HMGB1, which is found between tumor-infiltrating dendritic cells and TIM-3, inhibits the activity of innate immune response within the tumor microenvironment [18,19,20].

T cell immunoreceptor with Ig and ITIM domain is envisaged as one of the most important negative immune checkpoints able to inhibit the immune response at multidirectional levels of its activity [21,22]. TIGIT belongs to the family of Poliovirus Receptor (PVR)-like proteins. It can directly suppress T cells through interaction with CD226 (DNAM-1), while by IL-10 release stimulation, it can indirectly inhibit not only T cells but also other immune cells in the tumor microenvironment. High TIGIT expression on T cells can also negatively modulate NK cell function [23,24].

It can be noted that molecules expressed on immune cells involved in mediating innate responses should be considered as a target for immunotherapy. Macrophages seem to be greatly underestimated in the modulation of antitumor response; these cells can not only mediate inflammation during infection but promote immune escape within tumor microenvironments [25]. The new approach in immunotherapy is to block one of the human leukocyte immunoglobulin-like receptor B2 (LILRB2) family members (also known as ILT, LIR, and CD85), which is expressed by macrophages and is a negative regulator of myeloid cells. The mechanism of LILRB2 action is to compete with CD8 molecules expressed on T cells for MHC class I binding to modulate cytotoxic T cell action. This interaction ultimately leads to proinflammatory cytokines release suppression and promotes IL-10 and TGF-beta secretion [25].

## 5. Symbiotic Action between Negative and Positive Immune Checkpoints

The blocking function of negative immune checkpoints, in the form of monotherapy or in combination, is a well-known mechanism to reactivate exhausted immune cells. A new and very promising approach is to simultaneously block the negative immune checkpoints while stimulating (with agonistic antibodies) the positive immune checkpoints (costimulatory molecules). This should lead to unlocking inhibiting mechanisms and, on the other hand, exciting the cells’ engine to stimulate their action [26]. Within the group of positive immune checkpoints with strong costimulating properties, the following molecules were found:

CD137—a member of the tumor necrosis factor (TNF) receptor family, also named 4-1BB, functioning to upregulate TCR (T Cell Receptor)–ligand complex expression and stimulate cell proliferation and dendritic cell maturation [4,26].

ICOS (Inducible T cell COStimulator, CD278—a CD28-superfamily costimulatory molecule that is expressed on activated T cells. It plays a significant role in stimulating T cell proliferation and IL-2 production. Recent studies in animal models indicate that signaling through ICOS is of great importance in primarily enhancing the efficiency of CD8-positive T cells and promoting the production of tissue-resident T memory cells [27,28].

OX40—a molecule that is expressed on the surface of activated T lymphocytes, NK cells, and other immune cells involved in both adaptive and innate immunity. The interaction with its ligand, OX40L, is one of the most important survival signals for T cells. This interaction also promotes the development of T memory cells, which are responsible for the development of immunological memory and long-term survival observed in cancer patients undergoing immunotherapy [4,26].

**Figure 1:**
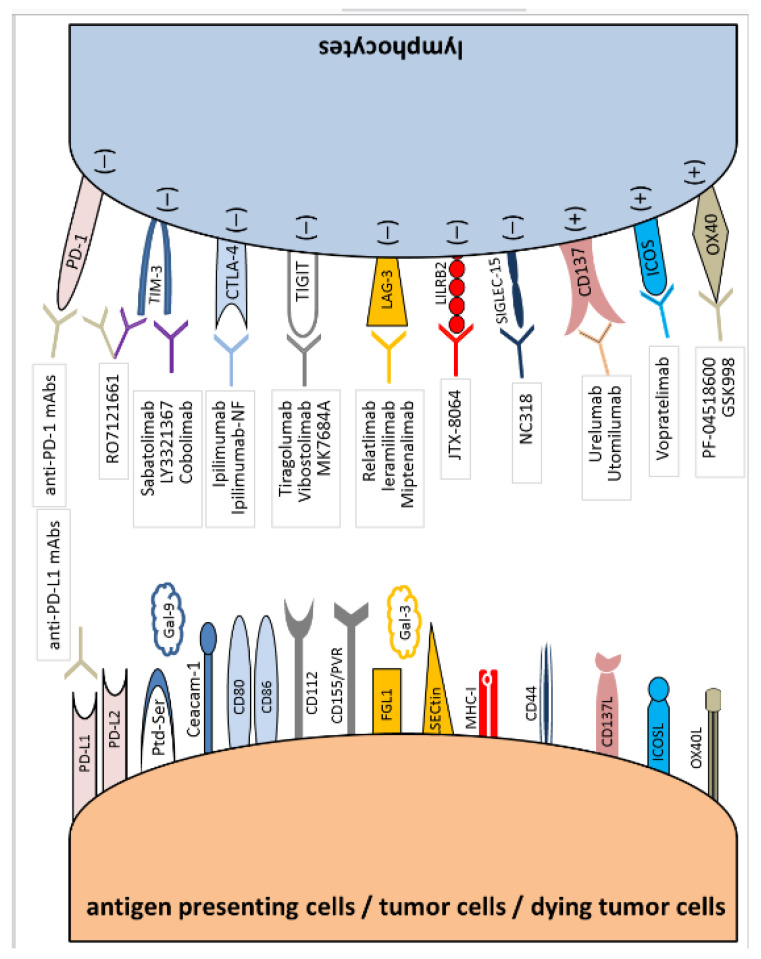
Immune checkpoint synapses with blocking agents attributed to specific receptors and ligands.

All interactions described in these chapters are shown in Figure 1.

## 6. Efficacy of Double Blockade in Immunotherapy-Resistant NSCLC Patients

### 6.1. Experience with Double Blockade of Immune Checkpoints in ICI-Naïve NSCLC Patients

The rationale for the use of a double blockade of immune checkpoints was provided by studies in which such treatment methods were used in NSCLC patients in the first line of treatment. In the CheckMate 227 study, the combined use of nivolumab and ipilimumab significantly increased the median OS, decreased the risk of death, and increased the percentage of patients with 4-year survival compared to chemotherapy in all groups of patients differing in the expression of PD-L1 on tumor cells. However, the efficacy of nivolumab in combination with ipilimumab and the effectiveness of nivolumab alone were only numerically higher. A total of 29% vs. 21% of patients with PD-L1 expression on ≥1% of tumor cells were alive after 4 years of observation in the group treated with the combination treatment and in the group receiving nivolumab monotherapy, respectively. In the group of patients with the expression of PD-L1 on ≥50% of cells, these percentages were as follows: 29% and 21%. ORR was 36% vs. 28% in this group of patients with any PD-L1 expression and was 45% vs. 37% in the group of patients with high PD-L1 expression. In the final publication, detailed data concerning safety were shown. Although many TRAEs were noted in the ipilimumab plus nivolumab arm during the treatment period, no new safety signals were seen for 2 years or more after immunotherapy cessation. TRAEs (any grade, grade 3 or more, respectively) in the ipilimumab plus nivolumab arm occurred in 76.7% and 32.8%, and they included diarrhea (17.2%, 1.7%), rashes (17.0%, 1.6%), pruritis (14.4%, 0.5%), fatigue (14.4%, 1.7%), decreased appetite (13.2%, 0.7%), hypothyroidism (12.5%, 0.3%), asthenia (10.2%, 1.4%), nausea (9.9%, 0.5%), vomiting (4.9%, 0.3%), constipation (4.5%, 0) and anemia (3.8%, 1.4%) as the most common ones. TRAEs leading to treatment discontinuation occurred in 18.1% of the immunotherapy combination arm, and 1.4% of treatment-related deaths were described, secondary to pneumonitis in 50% of the cases, and shock, myocarditis, acute tubular necrosis, and cardiac tamponade in the remaining patients. The manuscript also provides clinically important data about immune-related adverse events, their management, and outcome. The incidence of this typical toxicity was not showing new signals with hypersensitivity reactions within the first month of therapy, renal AEs, rash, and hyperthyroidism within the first 3 months, and a small number of patients experiencing new immunologic toxicity after 6–12 months of treatment. As clear immunologic toxicity management was implemented in the study, most of the immune TRAEs were resolved (74–100% depending on the event). The main therapy for immune TRAEs were systemic corticosteroids in the ipilimumab plus nivolumab arm given to 6% of patients with thyroid toxicity to 94% of patient with pneumonitis [29].

The phase 2 CITYSCAPE clinical trial showed significant progression-free survival and ORR benefits for the first-line combination of tiragolumab (anti-TIGIT antibody) and atezolizumab, compared to atezolizumab monotherapy in PD-L1 positive metastatic NSCLC patients. Median progression-free survival (mPFS) was 5.4 months in the patients treated with tiragolumab plus atezolizumab versus 3.6 months in patients who received atezolizumab monotherapy (HR = 0.57). A total of 31.3% and 16.2% of patients responded to combined therapy and to atezolizumab monotherapy, respectively. Particularly, a meaningful ORR improvement was seen in patients with expression of PD-L1 on ≥50% of tumor cells (55.2% vs. 17.2%). The reported safety profile of the combination arm showed a 9% incidence of grade 3 or worse lipase elevation and two treatment-related deaths secondary to pyrexia and infection [30].

The phase 3 studies SKYSCRAPER-01 (NCT04294810) and SKYSCRAPER-06 (NCT04619797) are underway. This clinical trial assesses the effectiveness of first-line therapy with tiragolumab plus atezolizumab vs. atezolizumab alone in 534 NSCLC patients with high PD-L1 expression as well as tiragolumab in combination with atezolizumab plus pemetrexed and platinum compound versus pembrolizumab plus platinum-based chemotherapy in 540 NSCLC patients regardless of PD-L1 expression. Moreover, the clinical trial using relatlimab (anti-LAG-3 antibody) in combination with nivolumab and chemotherapy compared to chemotherapy in combination with nivolumab used in the first line of treatment in NSCLC patients is currently recruiting (NCT04623775). The results of these studies seem promising [31]. 

Clinical trials with combined therapies in NSCLC patients with resistance to immunotherapy or chemoimmunotherapy with anti-PD-1 or anti-PD-L1 antibodies have been initiated based on the experience with the use of double blockade of immune checkpoints in the first line of treatment in NSCLC patients [1]. 

### 6.2. Blockade of PD-1 and CTLA-4 Molecules

The first trials involved ipilimumab in addition to nivolumab therapy. The phase II single-arm study NCT03262779 has been completed. This clinical trial evaluated the effectiveness of the addition of ipilimumab to nivolumab after primary resistance to anti-PD-1 therapy. The primary endpoint was an objective radiographic response, similar to the other studies described below. The investigators primarily enrolled patients who have experienced progression after anti-PD-1 therapy without initial response to such therapy. A smaller cohort of patients with acquired resistance to anti-PD-1 therapy was additionally included. The study involved 20 patients with advanced NSCLC. Unfortunately, the results of this study have not been reported [32]. 

The nonfucosylated (NF) version of ipilimumab (ipilimumab-NF, BMS-986118) has increased activity compared to ipilimumab, especially in the tumor environment. The nonfucosylated Fc region of ipilimumab has increased binding affinity to Fcγ receptors on neutrophils and NK cells and improves antibody-dependent cellular cytotoxicity (ADCC), thus increasing intratumoral Treg depletion. In NCT03110107, a phase I/II study, ipilimumab-NF was used alone or in combination with nivolumab in patients with advanced solid tumors. The study is still recruiting, and the number of patients who will be enrolled is 390. Lung adenocarcinoma or squamous cell carcinoma patients who have received standard therapies with proven survival benefits, including prior immunotherapy with anti-PD-1 or anti-PD-L1 antibodies, could participate in this study (Cohort 2B and 2C). Preliminary results on the safety of ipilimumab-NF have been published, but the effectiveness of the therapy is still being analyzed. Although most of the TRAEs were grade 1–2, they were observed in 52% of the treated patients, with pruritis and diarrhea as the most common ones. No grade 4 TRAE was observed, but in one patient, a fatal TRAE was reported secondary to pneumonitis. Treatment discontinuation caused by toxicity was experienced by 11% of patients [33,34]. 

### 6.3. Blockade of PD-1/PD-L1 Axis and TIM-3 Molecule

Several antagonistic antibodies to the TIM-3 molecule have been generated. Sabatolimab (MBG453) is a humanized IgG4 antibody that blocks TIM-3 and phosphatidylserine (PtdSer) ligand-dependent activation. In a phase II study (NCT02608268), sabatolimab in combination with spartalizumab (anti-PD-1 antibody) was used in patients with melanoma, renal cell carcinoma, or NSCLC who had progressive disease at or after therapy with anti-PD-1 or anti-PD-L1 antibodies. A total of 17 NSCLC patients, including 10 patients with durable clinical benefits in previous ICI therapy, received combined treatment with sabatolimab and spartalizumab. Seven patients achieved SD on combined therapy, including three patients with a prior long-term response to anti-PD-1 or anti-PD-1 monoclonal antibodies (mAbs), which indicates limited efficacy of therapy with sabatolimab and spartalizumab in ICI-refractory patients. The limited safety data available show that TRAEs occurred in 40% of patients treated with sabatolimab monotherapy (no grade 3–4 AEs) and in 59% of the group treated in combination with spartalizumab (11% grade 3–4 AEs), with fatigue as the most frequent AE in 10% and 15%, respectively [35]. 

Efficacy and safety of LY3321367 monotherapy or in combination with novel anti-PD-L1 antibody (LY3321367) were examined in a phase Ib clinical trial (NCT03099109) conducted in patients with advanced, treatment-refractory, solid tumors. LY3321367 is a humanized, IgG1, anti-TIM-3 antibody that inhibits both galactin-9 and PtdSer ligand-dependent activation. A total of 42 and 33 advanced NSCLC patients received LY3321367 monotherapy or combined treatment, respectively. Most patients had previously received prior ICI therapy with anti-PD-1 or anti-PD-L1 mAbs or combined treatment with anti-CTLA-4 and anti-PD-1 mAbs. In the dose escalation A cohort (monotherapy) and B cohort (combined treatment), no objective response was observed in NSCLC patients; however, two NSCLC patients achieved SD for more than 6 months. NSCLC patients in expansion cohort M (monotherapy) were divided into patients with confirmed progressive disease as the best response to prior immunotherapy (cohort M1) and patients who achieved a response or disease control to prior ICI therapy (cohort M2). In cohort M1, there was no response to therapy, and SD occurred in 34.8% of patients, while the mPFS was only 1.9 months. In the M2 cohort, there was one patient with a response to treatment (7.3%), disease stabilization was reported in 42.9% of patients, and the mPFS was 7.3 months. In NSCLC patients treated with the combination of LY3321367 and LY3300054 (cohort C), there was no response to immunotherapy, the percentage of patients with SD was 66.7%, and mPFS reached 3.7 months. The published results did not report any DLT. In the combination treatment dose escalation (B) and dose expansion (C) cohorts, several TRAEs have been observed. In cohort B, the most common TRAEs occurring in three patients or more included fatigue (14.3%), decreased appetite (10.7%), and infusion-related-reaction, while in cohort C, the TRAEs were fatigue (5.5%), rashes (4.4%), and increased AST (3.3%); all immune TRAEs in both cohorts were mild (grade 1–2) and had a good outcome. Although eight deaths were reported, none of them were found to be treatment-related [36]. 

TSR-022 (cobolimab) is a humanized anti-TIM-3 antibody of the IgG4 class. The phase II AMBER study (NCT02817633) evaluates the safety and efficacy of cobolimab alone or in combination with ICIs (anti-PD-1 mAbs: nivolumab or dostarlimab) or with cytostatics in patients with advanced solid tumors. The study consists of a dose escalation and dose expansion part. The first part included participants who received no more than two prior lines of therapies, which must include a platinum-based chemotherapy and an anti-PD-1 or anti-PD-L1 antibody. Such a group of patients is also enrolled in the second part of the study. Preliminary results in the group of patients treated with cobolimab in combination with nivolumab or dostarlimab from the first part of the study were reported in 2022. The ORR in patients receiving cobolimab and nivolumab was 42.9% (three out of seven patients). A total of 16.4% of patients responded to the combination of cobolimab and dostarlimab (9 out of 55 patients), and 29% of patients achieved SD. Data on the efficacy of combination therapy in NSCLC patients are not available. The study has the recruiting status, and the target number of patients with solid tumors is estimated at 475. In patients treated with the combination of nivolumab and cobolimab, the most common TRAEs of any grade were diarrhea (57.1%), nausea (42.9%), vomiting (42.9%), and fatigue (28.6%), with diarrhea (14.3%) and elevated ALT (14.3%) and elevated AST (14.3) as the most frequent grade 3–4 TRAEs. In patients treated with the combination of dostarlimab and all dosing levels of cobolimab, the most common TRAEs of any grade were fatigue (20.0%), rashes (14.5%), nausea (7.3%), diarrhea (7.3%), and vomiting (3.6%), with dyspnea (3.6%), diarrhea (3.6%), and rashes (3.6%) as the most frequent grade 3–4 TRAEs [37]. 

A phase I, multiple-ascending dose study (NCT03708328) of single-agent RO7121661 (anti-PD-1 and anti-TIM-3 bispecific antibody) for participants with advanced and metastatic solid tumors is underway. The study consists of two parts: dose escalation (Part A) and expansion (Parts B1, B2, B3, B4, and B5). In the dose expansion part of the study, NSCLC, PD-L1-positive patients previously treated with ICIs and platinum-based chemotherapy are enrolled (second or third line of RO7121661 therapy) [38]. 

### 6.4. Blockade of PD-1/PD-L1 Axis and TIGIT Molecule

The TIGIT molecule is the most caring therapeutic target for immunotherapy in cancer patients. This was proved, among others, by the results of the SKYSCRAPER studies. In patients with previously treated (including immunotherapy) advanced cancers, two clinical trials are currently recruiting patients for therapy with tiragolumab in combination with atezolizumab (anti-PD-L1 antibody). Tiragolumab (RG6058, MTIG7192A) is a humanized IgG1 monoclonal antibody that binds TIGIT to prevent its interaction with its ligand PVR (Poliovirus Receptor, CD155). The phase II study NCT04958811 is being conducted on 42 patients with advanced non-squamous NSCLC. Patients with PD-L1 expression on ≥1% of tumor cells receive a combination of three drugs: tiragolumab, atezolizumab, and bevacizumab. The criterion for inclusion in the study is the occurrence of disease progression during or following treatment with anti-PD-1 or anti-PD-L1 antibodies containing therapy [39]. The second study (NCT03977467, phase II) assesses the efficacy and safety of combining atezolizumab with standard-of-care chemotherapy in non-small cell lung cancer (NSCLC) and atezolizumab combined with tiragolumab in patients with advanced solid tumors who have progressed after prior exposure to anti-PD-1 or anti-PD-L1 treatment. Patients can cross over to the arm with atezolizumab and tiragolumab in the moment of progression during the treatment with atezolizumab and chemotherapy. Cohort B (combined immunotherapy) includes patients with advanced renal cell carcinoma progressing on anti-PD-1 therapy, advanced triple-negative breast cancer progressing on anti-PD-1 therapy, and patients with NSCLC progressing on checkpoint inhibitors plus chemotherapy in the first-line setting [40]. 

Vibostolimab (MK-7684) is humanized IgG1 antibody that blocks the TIGIT molecule. The phase I study NCT02964013 evaluated the safety, efficacy, and pharmacokinetic profiles of escalating doses of vibostolimab monotherapy or in combination with pembrolizumab in advanced NSCLC patients. The study involved 39 anti-PD-1 or anti-PD-L1-naïve patients (vibostolimab + pembrolizumab) and 67 anti-PD-1 or anti-PD-L1-refractory patients (34 patients received monotherapy and 33 patients received combined immunotherapy). In patients refractory to immunotherapy, vibostolimab alone resulted in one response (3%) and 10 disease stabilizations (29%), with a median duration of response (DoR) of 8.5 months. In ICI-resistant patients, 1 patient responded, and 14 patients achieved SD on vibostolimab in combination with pembrolizumab therapy with the median DoR not reached. The median PFS was 2 months (in both groups receiving monotherapy or combined treatment), and 10% of patients treated with vibostolimab as well as 25% of patients treated with vibostolimab and pembrolizumab survived 6 months without progression. PD-L1 expression seemed to have no effect on the efficacy of the vibostolimab monotherapy or vibostolimab plus pembrolizumab therapy in immunotherapy refractor patients. In the combination arm of the first part of the study, the vast majority of patients experienced AEs (95%), most commonly nausea and rashes. In terms of TRAEs, they were observed in 62% of the population, with pruritis and rashes as the most common, and with 17% of patients with reported TRAEs of grade 3–4, namely pruritis and rashes, adrenal insufficiency, and elevated ALT, and with 21% of the combination group with immune AEs and infusion reactions with no new immunological events. In the second part of the study, the majority of patients were treated with a combination of both immunotherapy-naïve and ant-PD-L1/PD-L1 pretreated, and safety was comparable in both cohorts, with 97% of the immunotherapy-naïve population experiencing any AE (most frequently pyrexia and hypoalbuminemia). Pruritus (38%) and hypoalbuminemia (31%) were the most common TRAEs [41,42]. 

A further clinical study with vibostolimab combination therapy is ongoing in patients with advanced therapy-refractory NSCLC. The efficacy of pembrolizumab and vibostolimab coformulation (MK-7684A) or pembrolizumab and vibostolimab coformulation plus docetaxel versus docetaxel is studied in metastatic NSCLC patients with progressive disease after platinum doublet chemotherapy and immunotherapy (NCT04725188, KEYVIBE-002). The recruited patients (*n* = 240) have progressive disease on treatment with one prior anti-PD-1 or anti-PD-L1 therapy administered either as a monotherapy or in combination with other checkpoint inhibitors or other therapies. Retreatment with the same anti-PD-L1 or anti-PD-L1 mAbs is acceptable in the overall course of treatment. Moreover, the study did not show any new safety signals for MK-7684A [43]. 

### 6.5. Blockade of PD-1/PD-L1 Axis and LAG-3 Molecule

Relatlimab (BMS-980016) is a first-in-class human IgG4 antibody against LAG-3 that restores the effector function of exhausted T cells. The phase II/III RELATIVITY-047 clinical trial showed a significant reduction in the risk of progression as a result of the use of relatlimab in combination with nivolumab compared to the use of anti-PD-1 mAb alone in patients with previously untreated metastatic or unresectable melanoma. The FDA has approved a new therapy for patients with melanoma. In the combination arm, the most common TRAE were pruritis, fatigue, rashes, arthralgia, hypothyroidism, diarrhea, and vitiligo, mostly of grades 1–2, with 18.9% of patients experiencing grade 3–4 AEs. Infusion-related AEs were observed in 5.9% of patients, while the most frequent grade 3–4 TRAEs in patients treated with relatlimab–nivolumab combination included increased activity of lipase, ALT, and AST. TRAEs leading to nivolumab–relatlimab discontinuation were observed in 14.6% of patients. Immune TRAEs were typical for immunotherapy, with no new safety signals. Myocarditis had a favorable outcome in all patients for the occurring AEs [44]. 

However, the FRACTION-Lung study (NCT02750514) using relatlimab and nivolumab in the treatment of advanced NSCLC patients (including immunotherapy-resistant patients) was terminated due to changes in the standard of care for this patients’ population. Among the 16 analyzed anti-PD-1 or anti-PD-L1-refractory patients, there was no response to treatment; only 1 patient completed the observation, and 13 patients experienced rapid disease progression [45]. 

*Ieramilimab (LAG525)* is another humanized IgG4 anti-LAG-3 mAb. In phase I/II of the NCT02460224 clinical trial, 235 patients were enrolled in the group that received combination treatment with ieramilimab plus spartalizumab. The majority (98%) had received prior antineoplastic therapy, including immunotherapy (28.6% of patients). A total of 42 NSCLC patients participated in this study, including 22 patients previously treated with anti-PD-1 or anti-PD-L1 therapy. No objective response to treatment with ieramilimab and spartalizumab was shown in pretreated NSCLC patients, but 50% of such patients achieved disease stabilization. The median PFS was 3.5 months in this group of patients [46,47]. As far as the combination arm safety profile is concerned, AEs of any grade were observed in 99.2% of patients with fatigue (36.4%), diarrhea (29.8%), decreased appetite (29.8%), constipation (24.0%), vomiting (23.1%), cough (23.1%), dyspnea (21.5%), and anemia (21.5%). Grade 3–4 AEs occurred in 54.5% of patients, but only 9.1% were treatment-related. Immune-related TRAEs included colitis, hepatitis, polyarthritis, and diabetic ketoacidosis. Treatment discontinuation took place in 4.1% of patients secondary to TRAE occurrence, namely colitis and diarrhea, brain tumor edema, pneumonitis, blurred vision, fatigue, and autoimmune hepatitis. None of the SAEs with fatal outcomes were found to be treatment-related [47]. 

Miptenalimab (*BI 754111*) is a humanized IgG4 anti-LAG3 monoclonal antibody. In the phase II study NCT03697304, miptenalimab was used in combination with the novel anti-PD-1 antibody ezabenlimab (BI 754091) in patients with anti-PD-1 or anti-PD-L1-naïve or pretreated metastatic solid tumors. This study was conducted based on the results of three phase I studies evaluating the pharmacodynamics, pharmacokinetics, and safety of miptenalimab and ezabenlimab combination doses in patients with advanced solid tumors (NCT03156114, NCT03433898, and NCT03780725). A cumulative analysis of the safety of these drugs was performed on 321 patients who participated in these four studies. However, data on the efficacy of miptenalimab in combination with ezabenlimab in patients with immunotherapy-resistant NSCLC are not yet available. The available results showed that the study therapy led to AEs of any grade in 91.3% of patients, namely fatigue, pyrexia, decreased appetite, nausea, diarrhea, vomiting, constipation, anemia, cough, and dyspnea, with grade 3 or more AEs in 32.1% of patients, such as anemia, dyspnea, fatigue. Study drug discontinuation occurred in 9.6% of patients, mainly due to AEs of grade 3 or more. All the deaths as a result of an AE were experienced by 1.2% of patients, and none of them was assessed as treatment-related [48,49]. 

### 6.6. Other Targets for Double Blockade

The INNATE phase I clinical trial (NCT04669899) evaluated JTX-8064 monotherapy (47 patients) and combination therapy with a new PD-1 inhibitor—pimivalimab (29 patients). JTX-8064 is a humanized IgG4 monoclonal antibody against macrophage leukocyte immunoglobulin-like receptor B2 (LILRB2/ILT4). This study enrolled advanced refractory solid tumor patients with progression on or after a prior PD-1 or PD-L1 inhibitor (with ICI primary or acquired resistance) and ICI-naïve patients. The results of the study were not published yet; thus, data concerning efficacy and safety are not available [50]. 

NC318 is a first-in-class humanized IgG1 monoclonal antibody that blocks S15-mediated immune suppression (SIGLEC-15). Phase I of the NCT03665285 dose-escalation study included patients with 15 tumor types (*n* = 49), and phase 2 (*n* = 47) included subjects with advanced solid tumors refractory or resistant to currently available therapies with the expression of PD-L1 on <50% of tumor cells. The median number of previous therapies was ≥3, including checkpoint inhibitors. The response was noted in two patients out of 13 NSCLC patients pretreated with ICIC therapy. High S15 expression on the tumor cell membrane was a predictor for stable disease and a longer duration of therapy. The data available for this trial only mention that NC318 was associated with good tolerance, and no new immunologic or safety signals were observed [51]. The phase II trial NCT04699123 is ongoing, which assesses the effectiveness of NC318 alone or in combination with pembrolizumab in patients with advanced NSCLC. A total of 141 patients have been recruited, including those with resistance to immunotherapy [52]. 

### 6.7. Efficacy and Safety of Agonistic and Antagonistic Antibody Targeted Immune Checkpoints in Immunotherapy-Resistant NSCLC Patients

In all of the clinical trials described above, immune checkpoint inhibitors were used in NSCLC patients and patients with solid tumors. However, there are agonists of immune checkpoints belonging to costimulatory molecules. Urelumab belongs to such agonistic antibodies by binding to the CD137 molecule. However, urelumab is characterized by high toxicity (especially hepatotoxicity). The new CD137 agonist is utomilumab. Utomilumab (PF-05082566) is a fully human IgG2 agonist mAb that selectively binds the human CD137 (4-1BB) molecule. In phase I of the NCT01307267 clinical trial, 43 patients with metastatic melanoma and 20 patients with advanced NSCLC, who had mostly progressed on prior ICIs, received utomilumab. Utomilumab in monotherapy did not show high efficacy (10 NSCLC patients with short-term SD) [53,54]. In the escalation cohort, no DLT was noted, but treatment-related AEs of any grade occurred in 34.5% of patients, with fatigue, pyrexia, decreased appetite, dizziness, and rashes occurring as the most frequent ones [53]. Utomilumab monotherapy led to grade 3–4 treatment-related AEs in 50.8% of patients, with fatigue, nausea, diarrhea, headache, and elevated AST as the most common ones. No treatment-related fatal AEs were observed, but serious treatment-related AEs occurred in 28.6% of patients, and in 7.9%, they led to permanent discontinuation due to cardio-respiratory arrest, colitis, enterocolitis, and hyperbilirubinemia [54].

Therefore, the phase Ib/II JAVELIN Medley study (NCT02554812) was designed, in which avelumab (an anti-PD-L1 antibody) was used in combination with the immune checkpoint agonists utomilumab and PF-04518600 (fully human IgG2 agonistic mAb specific for human OX40 on activated T cells) in patients with advanced malignancies. In cohorts A8, A9, and A10, NSCLC patients were treated with a combination of avelumab and utomilumab in the first line of treatment. However, numerous advanced NSCLC patients (cohorts A1 to A3) received this combined treatment at the moment of progression after previous lines of treatment, including immunotherapy. Patients with selected tumor types treated with a combination of three immunotherapeutics (avelumab, utomilumab, and OX40 agonist) were enrolled to dose escalation cohorts. A total of 398 patients were included in this clinical trial, but results for a previously treated NSCLC patient have not been posted [55].

Another target for immunotherapy is the activation of the costimulatory molecule, ICOS, on T lymphocytes. Vopratelimab (JTX-2011) is a first-in-class humanized IgG1 agonist antibody that specifically binds to ICOS. In the ICONIC phase I/II clinical trial (NCT02904226), vopratelimab was used as a monotherapy or in combination with nivolumab in patients with solid tumors with the expression of ICOS on CD4-positive T cells. The study involved 201 patients, including 70 patients treated with monotherapy and 131 patients with a combination of therapies. A total of 32 patients treated with vopratelimab alone and 67 patients treated with vopratelimab in combination with nivolumab received prior immunotherapy. In the immunotherapy-resistant group, 20% of patients in the first arm and 19.1% of patients in the second arm responded to treatment. There were 7 NSCLC patients who received vopratelimab and 17 NSCLC patients who were treated with combined therapy. No responses were observed in NSCLC patients, and 29.4% of patients experienced disease stabilization after vopratelimab together with nivolumab administration. The median PFS and OS in this second group of patients were 1.9 and 12.3 months, respectively. The mOS in groups that received combined treatment was significantly higher than in patients who received monotherapy (3.4 months). Vopratelimab monotherapy did not lead to limiting toxicity, with 56% of patients having grade 3–4 treatment-emergent AEs, most commonly including anemia, hyponatremia, diarrhea, elevated AST, and hyperbilirubinemia. Patients treated with the combination of nivolumab and vopratelimab experienced grade 3–4 treatment-emergent AEs in 48%, most commonly hyponatremia, anemia, elevated AST, and hyperbilirubinemia [56].

Vopratelimab is also being assessed in the SELECT phase II clinical trial (NCT04549025) in combination with pimivalimab in patients with metastatic NSCLC who are ICI-naïve and have progressed on platinum-based chemotherapy [57].

ENGAGE-1 (NCT02528357) is a phase I dose-escalation study evaluating the safety and effectiveness of GSK998 (humanized IgG1 mAb that targets and stimulates OX40 on T cells) alone and in combination with pembrolizumab in patients with previously treated advanced solid tumors, including NSCLC. A total of 138 patients were enrolled (45 patients were treated with GSK998 monotherapy, and 96 patients received GSK998 and pembrolizumab; crossover from the first group to the second group was allowed). Partial remission and stabilization of the disease lasting ≥24 weeks were observed in both anti-PD-1 or anti-PD-L1-naïve and ICI-refractory patients. In the monotherapy group, one patient with PR and one patient with SD were observed. However, two complete remissions, seven PRs, and nine SDs were shown in the group treated with GSK998 and pembrolizumab. Unfortunately, data regarding NSCLC patients and ICI-refractory patients are invisible. The study reported treatment-related AEs in 51% of patients with the most frequent fatigue and diarrhea. The only grade 3 AEs were lymphopenia, infusion-related reactions, and thrombocytopenia, with no grade 4 or fatal AEs and no DLT in Part 1 of the study. In Part 2, fatigue and nausea occurred in more than 10% of patients, and four events were grade 3. No grade 4 fatal AEs were noted, but in three patients, therapy was discontinued due to infusion-related reaction, pleural effusion, and grade 1 myocarditis with grade 3 troponin elevation [58].

One clinical trial attempted to overcome resistance to immunotherapy with two agonist antibodies, anti-OX40 (PF-04518600) and anti-CD137 (utomilumab). The phase I clinical trial NCT02315066 enrolled 20 NSCLC patients and 10 melanoma patients pretreated with ICIs. In NSCLC patients, objective responses to therapy occurred in 5% of patients, and stable disease occurred in 35% of patients. In terms of toxicity, the dose escalation regimen was associated with grade 1–2 AEs in 40.4% of patients and with grade 3–4 AEs in 49.1% of patients. Despite the fact that grade 5 AEs occurred in 10.5% of patients, none of them were associated with drug toxicity. Only 7% of patients discontinued therapy permanently (disease-related pain, hepatobiliary disorder, infusion-related reaction, and colon perforation). Generally, the safety profile of the combination of utomilumab and ivuxolimab was acceptable, with the treatment-related AEs occurring in >10% of patients, those being pruritus, anemia, fatigue, decreased appetite, and rashes. No serious treatment-related or leading-to-death AEs were observed [59].

All the discussed studies are summarized in Table 1.

## 7. Conclusions

The dual immunological checkpoint blockade remains a promising tool in optimizing the treatment of NSCLC patients with specific expectations in immunotherapy resistance management. Different combinations have been tested in phase I, II, and III trials, providing evidence that should drive future trial design and finally help clinicians to make optimal treatment decisions. Safety data on the combination approach show a variable benefit but, importantly, the safety profile seems promising and is generally well known based on phase III studies in NSCLC and other solid tumors. The oncologic community has integrated toxicity-management algorithms in daily practice, which helped to decrease treatment deaths even in phase I immunologic combination trials. These findings support further development of the dual blockade strategy and leave the possibility of incorporating new combinations into trials in order to find the optimal efficacy–safety balance in NSCLC patients.

## Figures and Tables

**Table 1:** Clinical trials dedicated to evaluating the safety and efficacy of combination therapy with immune checkpoint inhibitors and agonists in cancer patients. Columns 8–12 present the efficacy of combination immunotherapy in NSCLC patients refractory to ICIs.

Trial Name and Code	Phase	Status	Conditions	No of Patients	Line of Treatment	Interventions	ORR (n,%)	DCR (n,%)	Median DoR (mo)	Median PFS (mo)	Median OS (mo)
NCT03262779	II	Completed	NSCLC	20	1 and ≥2	− Nivolumab (anti-PD-1) − Ipilimumab (anti-CTLA-4)	No results posted				
NCT03110107	I/IIa	Recruiting	Advanced cancer	390	>2	− Nivolumab (anti-PD-1) − Ipilimumab NF (anti-CTLA-4)	No results posted				
NCT02608268	I-Ib/II	Terminated (business reason)	Advanced malignancies	252	≥2	− Sabatolimab (anti-TIM-3) − Spartalizumab (anti-PD-1)	0/17	7/17, 46.7%	-	-	-
NCT03099109	Ia/Ib	Active, not recruiting	Solid tumors	275	≥2	− LY3321367 (anti-TIM-3) − LY3321367 (anti-PD-1)	Cohort C2 (combined therapy)				
							0/21	14/21, 66.7%	-	3.7	-
							Cohort M1 (monotherapy with LY3321367, no response to prior ICI therapy)				
							0/21	8/23, 34.8%	-	1.9	-
							Cohort M2 (monotherapy with LY3321367, response to prior ICI therapy)				
							1/14, 7.1%	7/14, 50.0%	-	7.3	-
NCT02817633 AMBER	I	Recruiting	Advanced solid tumors	475	>2	− Cobolimab (anti-TIM-3) − Nivolumab or dostarlimab (anti-PD-1)	Cobolimab plus nivolumab				
							3/7, 42.9%	3/7, 42.9%	-	-	-
							Cobolimab plus dostarlimab (including NSCLC, skin cancer, and mesothelioma)				
							9/55, 16.4%	25/50, 50.0%	-	-	-
NCT03708328	I	Active, not recruiting	Advanced solid tumors	134	≥2	− RO7121661 (anti-PD-1 and anti-TIM3	No results posted				
NCT04958811	II	Recruiting	Non-squamous NSCLC	42	≥2	− Tiragolumab (anti-TIGIT) − Atezolizumab (anti-PD-L1) − Bevacizumab (anti-VEGF)	No results posted				
NCT03977467	II	Recruiting	Advanced solid tumors	80	≥2	− Tiragolumab (anti-TIGIT) − Atezolizumab (anti-PD-L1)	No results posted				
NCT02964013 KEYVIBE-001	I	Active, not recruiting	Advanced solid tumors	492	1 (with CTH) and ≥2	− Vibostolimab (anti-TIGIT) − Pembrolizumab (anti-PD-1)	Vibostolimab monotherapy				
							1/34, 2.9%	11/34, 32.4%	8.5	2.0	-
							Vibostolimab plus pembrolizumab				
							1/33, 3.0%	13/33, 45.4%	NR	2.0	-
NCT04725188 KEYVIBE-002	II	Active, not recruiting	Metastatic NSCLC	240	≥2	− Vibostolimab (anti-TIGIT) and pembrolizumab (anti-PD-1) coformulation vs. − Docetaxel	No results posted				
NCT02750514 FRACTION-Lung	II	Terminated (changes in SoC)	Advanced NSCLC	295	1 (different therapies) and ≥2	− Relatlimab (anti-LAG-3) − Nivolumab (anti-PD-1)	1/16 (6.25%) patients completed observation before study termination, and 13/16 (81.25%) patients with PD				
NCT02460224	I/II	Completed	Advanced malignancies	490	≥2	− Ieramilimab (anti-LAG-3) − Spartalizumab (anti-PD-1)	0/22	11/22, 50.0%		3.5	-
NCT03697304	II	Active, not recruiting	Advanced solid tumors	212	≥2	− Miptenalimab (anti-LAG-3) − Ezabenlimab (anti-PD-1)	No results posted				
NCT03156114	I	Active, not recruiting	Advanced cancer	172	≥2	− Miptenalimab (anti-LAG-3) − Ezabenlimab (anti-PD-1)	No results posted				
NCT03433898	I	Active, not recruiting	Asian patients with different types of cancer	146	≥2	− Miptenalimab (anti-LAG-3) − Ezabenlimab (anti-PD-1)	No results posted				
NCT03780725	I	Terminated due to company decision	NSCLC, HNSCC	8	≥2	− Miptenalimab (anti-LAG-3) − 89ZR-miptenalimab − Ezabenlimab (anti-PD-1)	No results posted				
NCT04669899 INNATE	I/II	Recruiting	Advanced refractory solid tumors	281	≥2	− JTX-8064 (anti-LILRB2/anti-ILT4) − Pimivatalimab (anti-PD-1)	No results posted				
NCT03665285	I/II	Active, not recruiting	SIGLEC-positive advanced solid tumors	109	≥3	− NC318 (anti-SIGLEC-15)	2/13, 15.4%	-	-	-	-
NCT04699123	II	Recruiting	Advanced NSCLC	141	≥2	− NC318 (anti-SIGLEC-15) − Pembrolizumab (anti-PD-1)	No results posted				
NCT01307267	I	Completed	Advanced cancer	190	≥2	− Utolimumab (CD137 agonist)	0/20	10/20, 50.0%	-	3.6	-
NCT02554812JAVELIN Medley	Ib/II	Active, not recruiting	Advanced malignancies	398	1 (different combined therapies) and ≥2	− Utolimumab (CD137 agonist) − PF-04518600 (OX40 agonist) − Avelumab (anti-PD-L1)	No results posted				
NCT02904226 ICONIC	I/II	Completed	Advanced and/or refractory solid tumors	242	1 (different combined therapies) and ≥2	− Vopratelimab (ICOS agnost) − Nivolumab (anti-PD-1) − Pembrolizumab (anti-PD-1) − Ipilimumab (anti-CTLA-4)	Vopratelimab monotherapy				
							0/7	0/7	-	1.9	3.4
							Vopratelimab plus nivolumab				
							0/17	5/17, 12.5%	-	1.9	12.3
NCT02528357 ENGAGE-1	I	Completed	Advanced solid tumor	141	≥2	− GSK3174998 (OX40 agonist) − Pembrolizumab (anti-PD-1)	No results posted (regarding ICI-refractory NSCLC patients)				
NCT02315066	I	Completed	Locally advanced or metastatic cancer	174	≥2	− PF-04518600 (OX40 agonist) − Utolimumab (CD137 agonist)	1/20, 5.0%	8/20, 40.0%	5.6	-	-
